# Effect of TAAR1/5-HT_1A_ agonist SEP-363856 on REM sleep in humans

**DOI:** 10.1038/s41398-021-01331-9

**Published:** 2021-04-20

**Authors:** Seth C. Hopkins, Nina Dedic, Kenneth S. Koblan

**Affiliations:** Sunovion Pharmaceuticals Inc, Marlborough, MA USA

**Keywords:** Molecular neuroscience, Prognostic markers

## Abstract

SEP-363856 is a trace amine-associated receptor 1 (TAAR1) and 5-hydroxytryptamine type 1A (5-HT_1A_) agonist, currently in Phase 3 clinical trials for the treatment of schizophrenia. Although SEP-363856 activates TAAR1 and 5-HT_1A_ receptors in vitro, an accessible marker of time- and concentration-dependent effects of SEP-363856 in humans is lacking. In rodents, SEP-363856 has been shown to suppress rapid eye movement (REM) sleep. The aim of the current study was to translate the REM sleep effects to humans and determine pharmacokinetic/pharmacodynamic (PK/PD) relationships of SEP-363856 on a measure of brain activity. The effects of SEP-363856 were evaluated in a randomized, double-blind, placebo-controlled, 2-way crossover study of single oral doses (50 and 10 mg) on REM sleep in healthy male subjects (*N* = 12 at each dose level). Drug concentrations were sampled during sleep to interpolate individual subject’s pharmacokinetic trajectories. SEP-363856 suppressed REM sleep parameters with very large effect sizes (>3) following single doses of 50 mg and plasma concentrations ≥100 ng/mL. Below that effective concentration, the 10 mg dose elicited much smaller effects, increasing only the latency to REM sleep (effect size = 1). The PK/PD relationships demonstrated that REM sleep probability increased as drug concentrations declined below 100 ng/mL over the course of the night. SEP-363856 was generally safe and well tolerated at both doses. The REM sleep-suppressing effects of SEP-363856 provide an accessible marker of brain activity, which can aid in dose selection and help elucidate its therapeutic potential in further clinical trials.

## Introduction

SEP-363856 is a novel central nervous system (CNS) active compound that has demonstrated significant efficacy in the treatment of the symptoms of acute schizophrenia in a large randomized, double-blind, placebo-controlled trial^1^. In contrast to all currently marketed antipsychotics, SEP-363856 does not act through dopamine D_2_ or 5-HT_2A_ receptors. Although its mechanism of action is not fully elucidated, preclinical studies suggest that agonism at TAAR1 and 5-HT_1A_ receptors contribute to its efficacy^2^. SEP-363856 has been shown to modulate dopaminergic and serotonergic neurotransmission through TAAR1- and 5-HT_1A_-mediated inhibition of dorsal raphe nucleus (DRN) and ventral tegmental area (VTA) neuronal firing^2^. In addition, SEP-363856 attenuates ketamine-induced increases in striatal dopamine synthesis capacity in mice, suggesting potential modulation of presynaptic dopamine dysfunction observed in schizophrenia patients^3^.

TAAR1 is a G-protein-coupled receptor that is expressed in cortical, limbic, and midbrain monoaminergic regions and has been shown to modulate dopaminergic, serotonergic, and glutamatergic activity in rodents^4–9^. Based on several preclinical studies with selective TAAR1 agonists^10–12^, and the recent clinical findings with SEP-363856^1^, TAAR1 has emerged as a promising therapeutic target for mental illness, addiction, and sleep disorders.

In a series of rodent and nonhuman primate studies, TAAR1 agonists have demonstrated wake-promoting effects and have been shown to reduce both rapid eye movement (REM) and non-rapid eye movement (NREM) sleep, likely via modulation of dopaminergic and serotonergic activity in regions associated with sleep state regulation^8,13–15^. These effects were TAAR1 dependent, as they were not observed in TAAR1 knockout mice, while wake promotion and REM suppression was strongly potentiated in TAAR1 overexpressing animals^14,16^. To date, studies assessing the effects of TAAR1 agonism on REM sleep in humans have not been published yet. In contrast, 5-HT_1A_ agonists have repeatedly been associated with REM suppression in healthy volunteers^17–22^.

In line with observations of TAAR1 and 5-HT_1A_ agonists, SEP-363856 was shown to robustly suppress REM sleep and increase the latency to REM in rats^2^. The objective of the current study was to evaluate the effect of single oral doses of SEP-363856 on REM sleep in healthy male subjects and characterize plasma concentration-response relationships to assist in dose selection for further evaluation in patients with schizophrenia.

## Methods and materials

### Subjects

The study enrolled healthy male subjects 18–35 years old (inclusive) with a body mass index (BMI) of 16-32 kg/m^2^. Enrollment was limited to subjects with no symptoms or history of a sleep disorder, and with normal sleep architecture based on the screening polysomnography (PSG). Wrist actigraphy was utilized to evaluate normal individual sleep and wake patterns to confirm eligibility. History of a psychiatric or neurologic diagnosis or any other current medical illness was reason for exclusion. Screening physical examination, electrocardiogram (ECG), and routine laboratory examinations (chemistries and hematologies) were required for study inclusion and also prior to randomization. During the study, treatment with any prescription or nonprescription drugs, vitamins, or dietary or herbal supplements was not permitted for at least 14 days prior to screening (or longer if the elimination half-life was known to be ≥150 h). In addition, subjects were not permitted to use alcohol, acetaminophen, or any caffeinated products within 48 h of a PSG (excluding screening PSG). Subjects were also not permitted to smoke, or use any tobacco (or nicotine products for smoking cessation) for the duration of the study; and must have refrained from strenuous exercise from the time of the screening physical examination until the end of the study. Subjects must have refrained from consuming citrus fruit juices within 7 days prior to the first dose of study drug through Day 11.

### Study design

**Fig. 1:**
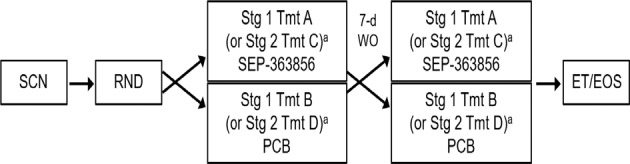
Study flow chart. *d* day, *EOS* end of study, *ET* early termination, *PCB* placebo, *RND* randomization, *SCN* screening, *Stg* stage, *Tmt* treatment, *V* visit, *WO* washout. ^a^Stage 1 (Group 1) preceded Stage 2 (Group 2).

This was a single-center, randomized, double-blind, placebo-controlled, 2-way crossover PSG study of single doses of SEP-363856 conducted in healthy male subjects (Fig. 1). The crossover study was designed for testing in 2 separate stages, one for a dose of SEP-363856 able to match plasma levels for the REM effect observed in animal studies (50 mg was selected) and one for a lower/higher dose level depending on the results observed at the first dose level (10 mg was selected). To ensure the double-blind, and to correct for any possible order effect, each subject was randomly assigned to one of two treatment sequences for each of the two dosages (50 mg or 10 mg) of SEP-363856 (sequence A: active drug followed by crossover to placebo; or sequence B: placebo followed by crossover to the active drug). A total of 12 subjects were enrolled at each SEP-363856 dosage, 6 subjects in treatment sequence A, and 6 in sequence B.

The flow of study activities was as follows: the first screening visit occurred 5-30 days before randomization. The subjects came into the clinic on Day 1 to confirm their eligibility, and to enter the initial phase of the crossover study. Dinner was provided approximately 4 h prior to lights out on each PSG testing night. On Night 1 baseline (adaptation) PSG was performed. Subjects remained in the clinic the next day. Experimental (on study drug) PSG was performed on Night 2, after study drug was administered approximately 15 min before lights out. Blood was obtained for pharmacokinetic (PK) assessments via an indwelling catheter at pre-dose baseline, and at 2, 4, 6, and 8 h post-dose. Subjects were discharged home on Day 3 to begin a ≥ 7-day washout period. Subjects then returned to the clinic for the second phase of the crossover study that repeated the same sequence of study events.

The study was approved by an institutional review board at the investigational site and was conducted in accordance with the International Conference on Harmonisation Good Clinical Practices guidelines and with the ethical principles of the Declaration of Helsinki. All subjects who entered the study reviewed and signed an informed consent document explaining study procedures and potential risks before study entry and before screening procedures were begun on Day 1.

### Polysomnography

PSG recordings were made using Grael 4K PSG:EEG equipment (Compumedics, Charlotte, NC). Silver-silver chloride electrodes were placed according to the International 10–20 system and the recommendations of Rechtschaffen and Kales^23^ to ensure standardization, and included EEGs, electrooculograms (EOGs), chin electromyograms (EMGs), 5-lead ECGs, leg EMGs and respiratory monitoring was performed using respiratory bands. PSG data were analyzed and scored according to Rechtschaffen and Kales^23^ criteria by an experienced sleep scorer blind to treatment condition. The primary endpoint was REM suppression as measured by change from baseline to post-dose timepoints in the following PSG measures: (1) REM latency (defined as duration in minutes from the first epoch of LPS to the first epoch of REM sleep; (2) REM duration in minutes; and (3) REM percent (defined as the number of minutes scored as REM sleep divided by total sleep time). Secondary endpoints consisted of the following measures derived from the PSG data: total sleep time (TST); latency to persistent sleep (LPS); NREM duration in minutes and NREM percent (defined as the number of minutes scored as NREM sleep divided by total sleep time); minutes of wake time after sleep onset (WASO); and sleep efficiency (SE; defined as the ratio of total sleep time to the time in bed).

### Pharmacokinetic measurements

Blood samples for determination of plasma concentrations of SEP-363856 were collected pre-dose on Day 1, and at 2, 4, 6, and 8 h post-dose on Days 2 and 10. Individual SEP-363856 plasma concentrations were determined by HPLC/MS/MS to estimate PK parameters Cmax, tmax, and AUC. Half-maximal SEP-363856 concentration effects on REM parameters were estimated by nonlinear regression. Concentration-time trajectories for individual subjects were computed by population PK methods.

### Safety

Safety assessments included treatment-emergent and serious adverse events (TEAEs and SAEs), physical examination and brief neurological examination; vital signs; 12-lead ECG; and laboratory tests (chemistries, hematologies, urinalysis, coagulation indices, thyroid panel, lipid panel, prolactin). Suicidality was assessed using the Columbia–Suicide Severity Rating Scale (C-SSRS).

### Statistical analysis

Baseline data were presented for all subjects by each stage, by each treatment sequence, and by each treatment. Polysomnography parameters and safety measures were summarized by treatment using descriptive statistics. For continuous outcomes, descriptive statistics included the number of subjects, mean, 95% confidence interval (CI) for the mean, standard deviation (SD), median, minimum, and maximum. Effect sizes for discussion were calculated using mean differences between drug treatment and placebo post-baseline, divided by the pooled standard deviation of the drug and placebo groups (Cohen’s d). A linear mixed model analysis was performed for the 3 co-primary REM endpoints (REM latency, REM duration, REM percent) that evaluated LS mean (95% CI) differences between active treatment and placebo with treatment, treatment period, and treatment sequence as fixed effects, and subject nested within the sequence as a random effect. No adjustments for multiplicity were applied.

## Results

All 24 of the healthy male subjects randomized to crossover treatment with either the 50 mg or 10 mg doses of SEP-363856 (or placebo) completed the study. Baseline characteristics were similar for subjects in both the 50 mg and 10 mg stages, respectively (Table 1).

**Table 1:** Baseline characteristics of safety population.

	Stage 1^a^ (SEP-363856 50 mg vs. placebo) (*N* = 12)	Stage 2^a^ (SEP-363856 10 mg vs. placebo) (*N* = 12)
Age, mean (SD), years	25.3 (4.7)	24.9 (3.6)
Race, *n* (%)		
White	6 (50%)	6 (50%)
Black/African-American	6 (50%)	3 (25%)
Asian	0	3 (25%)
Hispanic/Latino, *n* (%)	2 (16.7%)	4 (33.3%)
Height, mean (SD), cm	174.6 (7.2)	175.9 (8.4)
Weight, mean (SD), kg	72.9 (12.0)	75.6 (11.8)
Body mass index, kg/m^2^	23.9 (3.7)	24.4 (3.3)

^a^Patients in each stage assigned to treatment sequence 1 (SEP-363856 → placebo; *n* = 6) and treatment sequence 2 (placebo → SEP-363856; *n* = 6) are combined.

### Sleep architecture

#### Effects on REM parameters

**Fig. 2:**
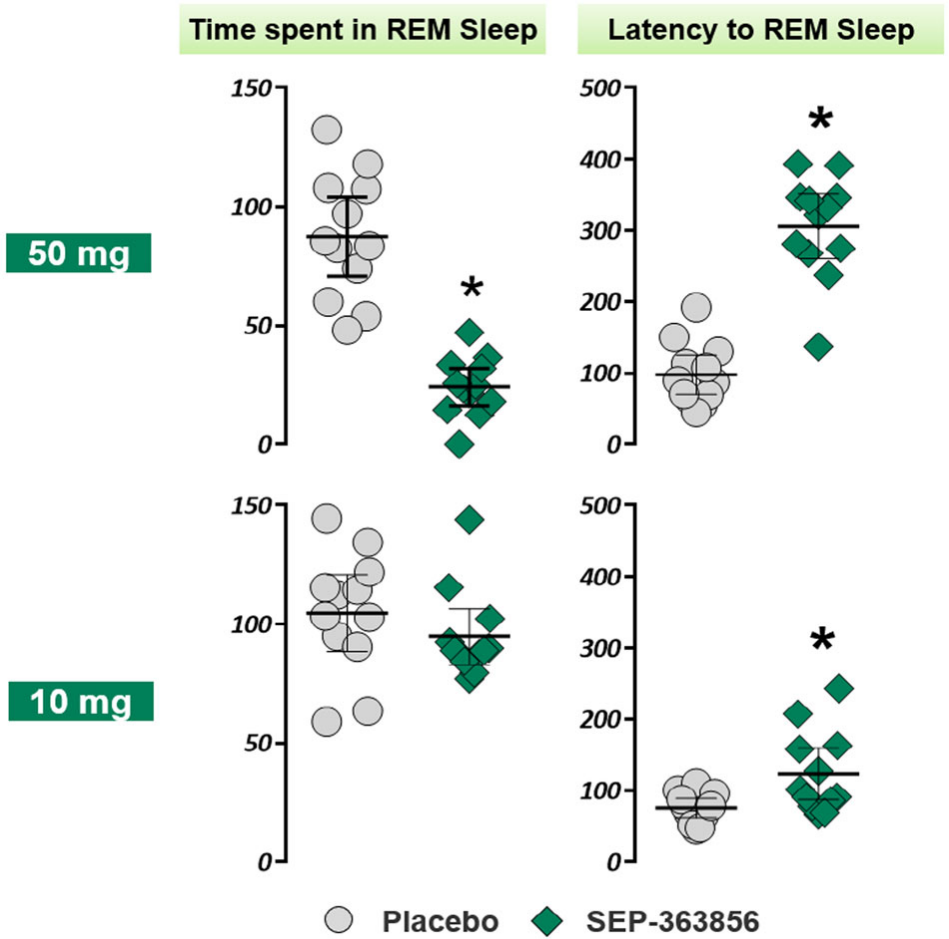
Time spent in REM sleep (minutes) and latency to REM sleep (minutes) for SEP-363856 vs. placebo. Post-baseline values are shown. *N* = 24 subjects. Mean ± 95% C.I. *P < 0.05, based on linear mixed model analysis.

**Fig. 3:**
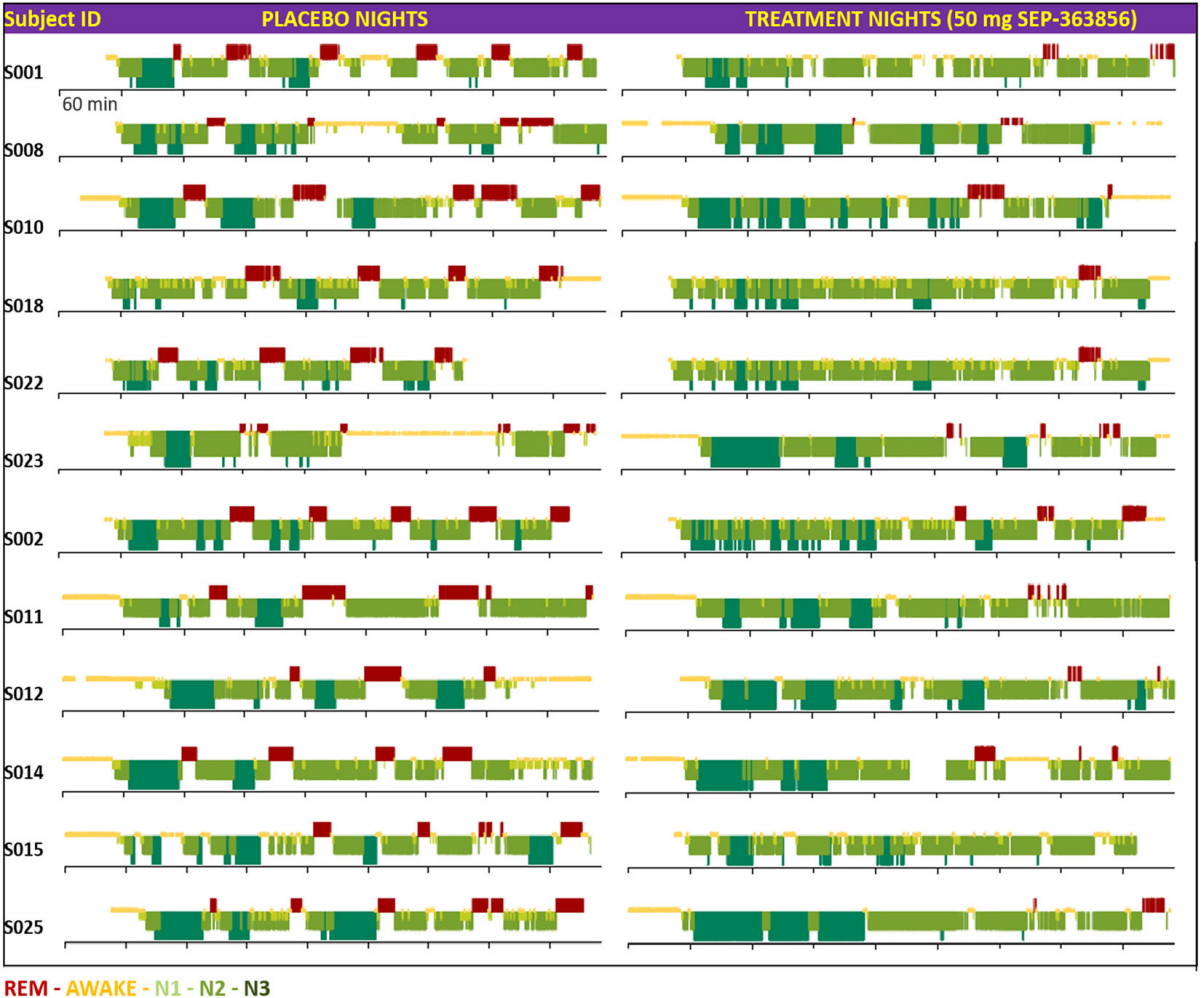
Individual hypnograms from all 12 subjects following crossover treatment with 50 mg SEP-363856 or placebo. SEP-363856 potently suppressed REM sleep and increased the latency to REM sleep across all subjects.

**Fig. 4:**
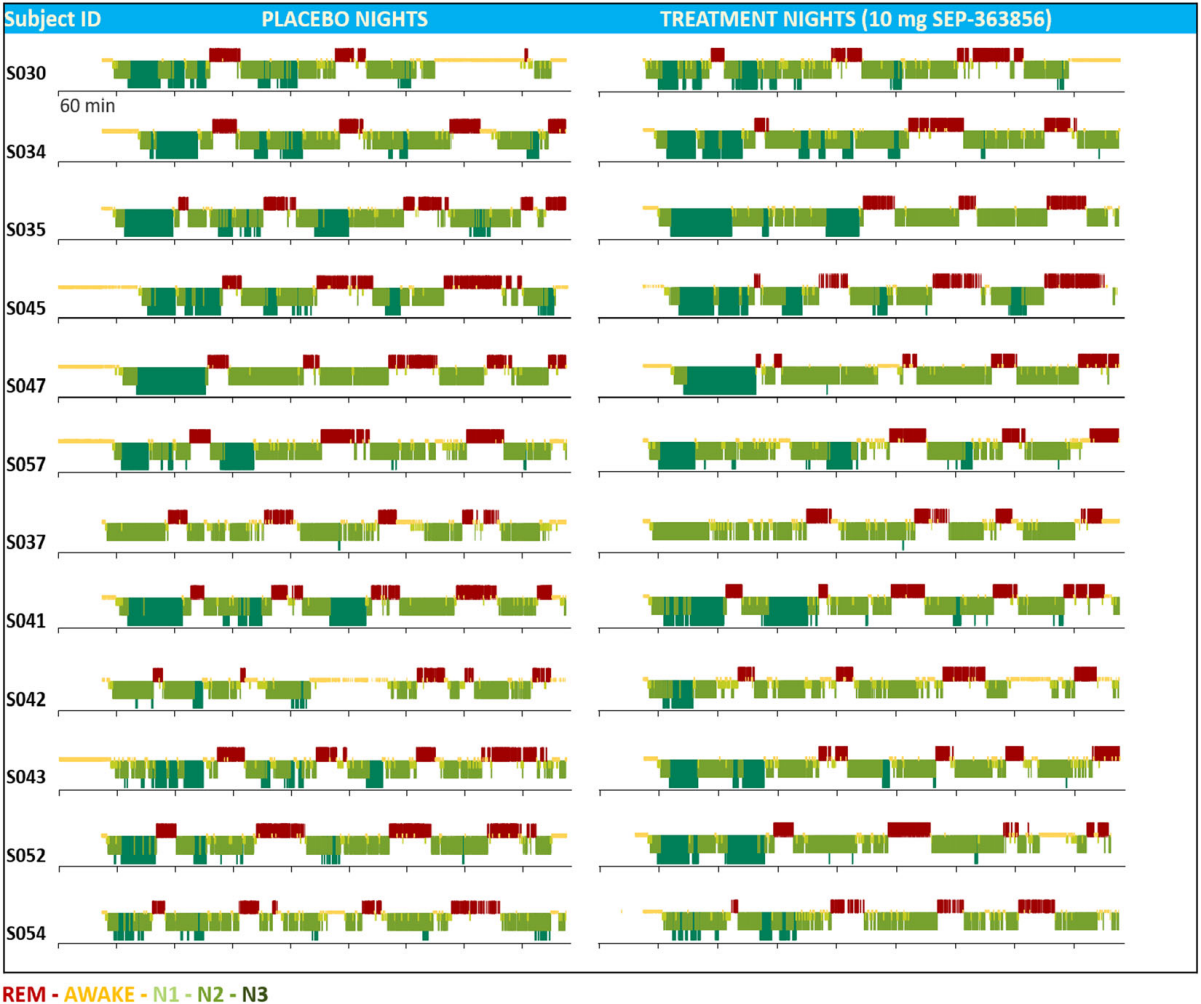
Individual hypnograms from all 12 subjects following crossover treatment with 10 mg SEP-363856 or placebo. SEP-363856 increased the latency to REM sleep in most subjects.

The single 50 mg dose of SEP-363856 was associated with a significant reduction versus placebo in REM duration (difference in LS mean change scores −52.4 min [95% CI: −72.2, −32.7]); REM percent (difference in LS mean change scores −13.4 [95% CI: −16.8, −9.9]); and significant increase in the latency to REM (difference in LS mean change scores +186.0 min [95% CI: 127.1, 244.9] (Table 2). The single 10 mg dose of SEP-363856 was associated with a non-significant reduction versus placebo in REM duration (difference in LS mean change scores −11.0 min [95% CI: −30.8, +8.7]) and REM percent (difference in LS mean change scores −2.3 [95% CI: −6.2, +1.6]); and significant increase in the latency to REM (difference in LS mean change scores +58.3 min [95% CI: 15.3, 101.4] (Table 2). Figure 2 displays the significant reduction in time spent in REM sleep, and the increase in REM latency after a single 50 mg dose of SEP-363856. The 10 mg dose was associated with a significant reduction in REM latency, but not in time spent in REM. Hypnograms for individual subjects show the striking REM suppressant effect of the 50 mg dose of SEP-363856 (Fig. 3), and the lesser REM suppressant effect of the 10 mg dose (Fig. 4).

**Table 2:** REM sleep parameters in healthy males (*N* = 12 per dose cohort) during crossover treatment with SEP-363856 (10 mg or 50 mg) vs. Placebo.

	Stage 1		Stage 2	
	SEP-363856 50 mg (*N* = 12)	Placebo (*N* = 12)	SEP-363856 10 mg (*N* = 12)	Placebo (*N* = 12)
**REM latency (min)**				
Baseline, mean (SD)	93.2 (55.6)	63.4 (12.8)	69.7 (13.5)	79.9 (23.1)
Post-baseline, mean (SD)	313.3 (84.6)	97.5 (43.3)	123.5 (57.6)	75.5 (21.1)
Change from Baseline, mean (SD; 95% CI)	+220.1 (83.4; 176.9, 263.4)	+34.1 (41.0; 12.9, 55.4)	+53.9 (65.5; 19.9, 87.8)	−4.5 (20.4; −15.0, 6.1)
LS mean difference score (95% CI)	186.0 (127.1, 244.9)*		58.3 (15.3, 101.4)*	
**REM time (min)**				
Baseline, mean (SD)	99.4 (24.2)	110.4 (23.2)	114.6 (16.6)	113.5 (26.5)
Post-baseline, mean (SD)	24.1 (12.4)	87.5 (26.2)	94.9 (18.5)	104.8 (25.5)
Change from Baseline, mean (SD; 95% CI)	−75.2 (19.6; −85.4, −65.1)	−22.8 (26.7; −36.7, −9.0)	−19.8 (19.0; −29.6, −9.9)	−8.7 (27.3; −22.9, 5.5)
LS mean difference score (95% CI)	−52.4 (−72.2, −32.7)*		−11.0 (−30.8, 8.7)	
**REM percent**				
Baseline, mean (SD)	23.8 (5.0)	25.9 (4.1)	26.6 (3.1)	26.9 (4.7)
Post-baseline, mean (SD)	5.9 (3.0)	21.4 (4.5)	22.4 (4.0)	25.0 (4.7)
Change from Baseline, mean (SD; 95% CI)	−17.9 (3.8; −19.8, −15.9)	−4.5 (4.9; −7.0, −2.0)	−4.2 (3.4; −6.0, −2.4)	−1.8 (5.3; −4.6, 0.9)
LS mean difference score (95% CI)	−13.4 (−16.8, −9.9)*		−2.3 (−6.2, 1.6)	

Latency to REM sleep was defined as duration in minutes from the first epoch of latency to persistent sleep to the first epoch of REM sleep. REM time was defined as total number of minutes scored as REM sleep. REM percent was defined as the number of minutes scored as REM sleep divided by total sleep time. Baseline for treatment period 1 was defined as measurement at Visit 2 Night 1; and baseline for treatment period 2 was defined as measurement at Visit 3 Night 9. Post-baseline was on dosing nights 2 and 10, respectively. The LS mean difference score was calculated as LS mean of SEP-363856 minus LS mean of placebo in change from baseline.

*CI* confidence interval, *LS mean* least squares mean, *REM* rapid eye movement, *SD* standard deviation.

*P < 0.05 based on a linear mixed model analysis.

#### Effects on non-REM parameters

The 50 mg dose of SEP-363856 was also associated with a small numerical increase compared with pre-treatment baseline in time spent in NREM stage 2 sleep, and in NREM stage 3 (slow wave) sleep. No treatment effect (compared with pre-treatment baseline) was observed on NREM stage 2 or stage 3 sleep for the 10 mg dose of SEP-363856 or placebo. No apparent differences were observed for either dose of SEP-363856 in total sleep time, sleep efficiency, LPS, WASO, and NREM stage 1 sleep (Table 3).

**Table 3:** Additional polysomnography parameters in healthy males (*N* = 12 per dose cohort) during crossover treatment with SEP-363856 (10 mg or 50 mg) vs. placebo.

	Stage 1		Stage 2	
	SEP-363856 50 mg (*N* = 12)	Placebo (*N* = 12)	SEP-363856 10 mg (*N* = 12)	Placebo (*N* = 12)
**Total sleep time (min)**				
Baseline, mean (SD)	415.0 (27.9)	423.8 (33.7)	430.4 (26.7)	419.4 (34.2)
Post-baseline, mean (SD)	409.7 (23.9)	402.1 (50.2)	422.8 (23.1)	414.4 (36.7)
Change from Baseline, mean (SD; 95% CI)	−5.3 (30.7; −21.2, 10.6)	−21.7 (55.8; −50.6, 7.3)	−7.5 (27.7; −21.9, 6.8)	−5.0 (30.8; −21.0, 11.0)
**Sleep efficiency (%)**				
Baseline, mean (SD)	86.5 (5.8)	88.3 (7.0)	89.7 (5.6)	87.4 (7.2)
Post-baseline, mean (SD)	85.4 (5.0)	83.8 (10.5)	88.1 (4.8)	86.3 (7.6)
Change from Baseline, mean (SD; 95% CI)	−1.1 (6.4; −4.4, 2.2)	−4.5 (11.7; −10.6, 1.5)	−1.6 (5.8; −4.6, 1.4)	−1.1 (6.4; −4.4, 2.2)
**LPS (min)**				
Baseline, mean (SD)	21.1 (22.7)	14.6 (14.4)	14.0 (11.7)	11.1 (8.3)
Post-baseline, mean (SD)	16.6 (12.0)	18.6 (16.3)	12.5 (7.4)	17.0 (13.1)
Change from Baseline, mean (SD; 95% CI)	−4.5 (21.3; −15.5, 6.5)	+4.0 (9.4; −0.9, 8.9)	−1.5 (9.7; −6.5, 3.5)	+5.9 (11.0; 0.2, 11.6)
**WASO (min)**				
Baseline, mean (SD)	53.7 (25.5)	43.6 (34.2)	37.8 (25.0)	48.5 (34.9)
Post-baseline, mean (SD)	55.42 (21.5)	63.7 (45.9)	45.21 (25.9)	51.63 (39.2)
Change from Baseline, mean (SD; 95% CI)	+1.7 (26.8; −12.2, 15.6)	+20.1 (57.2; −9.6, 49.7)	+7.4 (23.5; −4.8, 19.6)	+3.1 (33.7; −14.4, 20.6)
**NREM stage 1 (min)**				
Baseline, mean (SD)	38.4 (12.9)	36.1 (14.4)	32.5 (18.8)	37.1 (18.8)
Post-baseline, mean (SD)	32.8 (17.7)	35.9 (10.7)	33.9 (15.8)	34.6 (13.5)
Change from Baseline, mean (SD; 95% CI)	−5.6 (13.0; −12.4, 1.2)	−0.2 (17.3; −9.2, 8.7)	+1.4 (10.3; −4.0, 6.7)	−2.5 (13.6; −9.6, 4.6)
**NREM stage 2 (min)**				
Baseline, mean (SD)	239.9 (42.0)	232.3 (42.4)	213.8 (37.7)	203.6 (31.8)
Post-baseline, mean (SD)	265.2 (31.8)	214.4 (39.5)	224.7 (29.3)	204.6 (30.2)
Change from Baseline, mean (SD; 95% CI)	+25.2 (41.9; 3.6, 46.9)	−17.9 (59.7; −48.8, 13.1)	+10.9 (33.6; −6.5, 28.3)	+1.0 (16.6; −7.6, 9.6)
**NREM stage 3 (min)**				
Baseline, mean (SD)	37.3 (31.9)	45.0 (35.8)	69.4 (38.1)	65.1 (28.1)
Post-baseline, mean (SD)	87.6 (36.0)	64.3 (26.1)	69.3 (34.0)	70.4 (36.4)
Change from Baseline, mean (SD; 95% CI)	+50.3 (26.9; 36.4, 64.2)	+19.3 (35.6; 0.8, 37.7)	−0.04 (20.2; −10.5, 10.4)	+5.2 (14.3; −2.2, 12.7)

LPS was defined as duration in minutes from ‘lights out’ to the first epoch of 10 consecutive minutes of scoreable sleep. WASO was defined as the number of minutes of wake time following sleep onset. Baseline for treatment period 1 was defined as measurement at Visit 2 Night 1; and baseline for treatment period 2 was defined as measurement at Visit 3 Night 9. Post-baseline was on dosing nights 2 and 10, respectively. The LS mean difference score was calculated as LS mean of SEP-363856 minus LS mean of placebo in change from baseline.

*CI* confidence interval, *LPS* latency to persistent sleep, *LS mean* least squares mean, *REM* rapid eye movement, *NREM* non-REM, *SD* standard deviation, *WASO* wake after sleep onset.

### Pharmacokinetic assessments

**Fig. 5:**
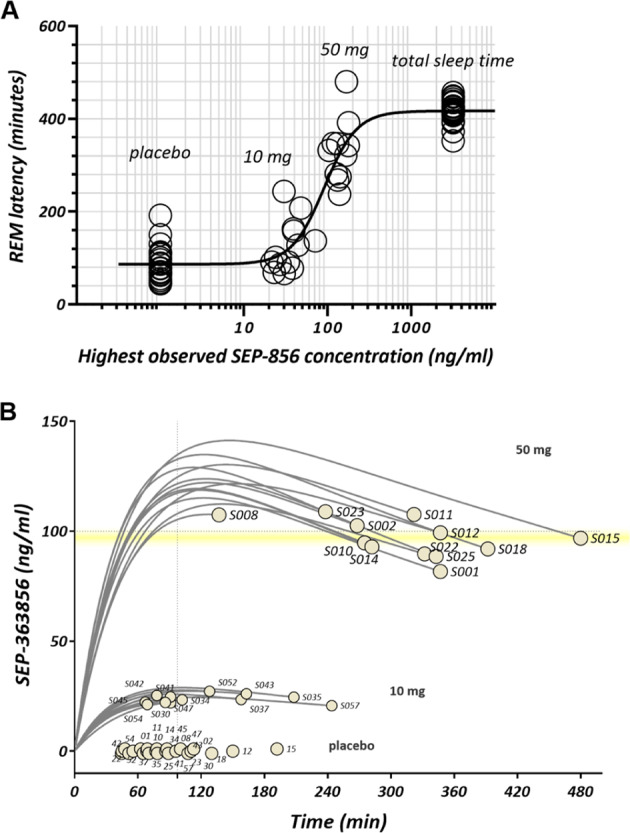
Effect of SEP-363856 plasma concentration on REM parameters. **A** Effects of SEP-363856 concentration on the latency to REM sleep. Nonlinear regression of all subjects’ placebo, total sleep time, and highest observed concentrations estimated half-maximal effects at 100 ng/mL. Placebo and total sleep time were assigned to minimum and maximum concentrations respectively. 95% C.I. of fitted EC50 = 77–110 ng/ml. **B** Concentration trajectories until first REM sleep episode. Circles represent the time of a labeled subjects first REM sleep episode. The trajectories represent the interpolated drug concentrations prior to the first REM sleep episode. Individual subject’s plasma samples (4 plasma samples per subject) were used together with a population-based pharmacokinetic model to interpolate trajectories for each subject shown (subject ID).

For SEP-363856 50 mg and 10 mg doses, respectively, the PK parameters were as follows for Cmax (mean, 140 and 33.7 ng/mL), tmax (median, 3.0 and 2.0 h), and AUC(0-8 h) (mean, 821 and 151 ng h/mL). Variability in exposure parameters for SEP-363856 was low to moderate with CV% values of 26.2% and 16.4% for Cmax and 22.2% and 14.0% for AUC(0–8 h) for the 10 and 50 mg doses, respectively. The half-maximal effect of SEP-363856 concentration on REM parameters was determined by the fitting time spent in REM (minutes) for all subjects and all doses. Figure 5A shows the effect of SEP-363856 concentration on REM latency, where placebo was assigned to the asymptote of lowest effect (on REM minutes for each placebo subject tested) and total sleep time was assigned to maximal effect (total sleep time for each subject tested). Nonlinear regression of all subjects’ placebo, total sleep time, and highest observed concentrations produced an estimate of 100 ng/mL for half-maximal effects. Separately, individual subjects’ concentration-time trajectories were computed by population PK methods to interpolate the plasma concentrations at which each subject’s REM episode occurred. Figure 5B shows that REM episodes occur at and below 100 ng/mL as the probability of REM suppression decreases over the 8 h sleep time.

### Safety

No subject had a severe TEAE, a treatment-emergent SAE or died, or discontinued the study due to a TEAE. The following individual AEs were reported by one subject in each study treatment as follows: SEP-363856 50 mg (enteritis, influenza, somnolence), SEP-363856 10 mg (nausea, infusion site hematoma), and placebo (pain in extremity, abnormal dreams). All TEAEs were considered either mild or moderate in severity. There were no clinically significant changes from baseline in vital signs, physical examination, laboratory values (including prolactin), or ECG parameters, including no QTcF values ≥450 milliseconds or an increase in QTcF ≥60 ms. No subjects exhibited any self-injurious behavior or suicidal ideation or behavior. Responses to the Columbia-Suicide Severity scale for subjects in each treatment group was negative. Overall, SEP-363856 was generally safe and well tolerated when administered as a single oral dose of 50 mg or 10 mg to healthy male subjects.

## Discussion

The current study assessed the effects of single oral doses of SEP-363856 (10 mg or 50 mg) on sleep architecture, particularly REM sleep, in healthy male subjects. In addition, the aim was to characterize the doses and plasma concentrations of SEP-363856 associated with clinically relevant CNS activity, and use REM effects as a translational pharmacodynamic measure to assist in dose selection for subsequent clinical trials in schizophrenia. The 50 mg dose of SEP-363856 produced a remarkably large suppression of REM sleep. REM sleep parameters changed with very large effect sizes (e.s.), as measured by increased latency to REM (e.s. 3.2) and decreased REM duration (e.s. 3.1) and percent (e.s. 4.1). At 10 mg, SEP-363856 increased REM latency (e.s. 1.1) but did not change REM duration and REM percent compared to placebo. In addition, single-dose treatment with 50 mg of SEP-363856 resulted in a small increase in NREM stage 2 and stage 3 sleep compared to placebo. No differences were observed on sleep continuity and other NREM sleep parameters, including total sleep time, for either dose of SEP-363856. Nonlinear regression modeling identified a SEP-363856 plasma concentration of 100 ng/mL as sufficient to achieve and maintain effects on REM sleep. The REM-suppressing effects were consistent with earlier results obtained in rats, and the plasma concentration was in the range previously associated with efficacy in animal models of psychosis^2^.

The large effect size for REM suppression enabled a precise depiction of time- and concentration-dependent drug effects on a directly observable, physiological, normal CNS function at the single-subject level. Examination of the placebo-treated subjects’ hypnograms demonstrates a physiological distribution of REM sleep episodes throughout a normal 8-h sleep cycle. The PK/PD relationship of SEP-363856 on REM episodes was remarkable at the level of the hypnograms (visually), at the level of interpolated drug concentrations (half-maximal effective concentration of 100 ng/mL) and at the level of trajectories prior to the first REM episode (REM episodes emerge as drug concentration falls below 100 ng/mL). Together the PK/PD findings from the current study guided dose-selection (50 and 75 mg) in a subsequent randomized, double-blind, 4-week clinical trial in patients with an acute exacerbation of schizophrenia^1^. In this trial, SEP-363856 demonstrated significant efficacy in reducing symptom severity compared to placebo as measured by the Positive and Negative Syndrome Scale (PANSS) total score. These results confirm the utility of REM sleep suppression as a translational pharmacodynamic measure for dose selection in clinical trials.

Several neuropsychiatric disorders, including schizophrenia, are associated with sleep disturbances and antipsychotic drugs exert varying effects on sleep^24–26^. The functional significance and clinical relevance of REM suppression across different psychiatric disorders are still largely unknown. To this point, it is currently unclear whether SEP-363856 effects on REM sleep are related to its clinical effects in schizophrenia patients. Notably, 4-week treatment with SEP-36856 (50 or 75 mg) in schizophrenia patients was associated with improved sleep quality compared to placebo, assessed with the Pittsburgh Sleep Quality Index^1^. However, it is important to point out that sleep quality was evaluated as part of the safety assessment and not included as a primary outcome measure of the study. Future clinical trials are required to confirm, and comprehensively assesses the effects of SEP-363856 on sleep quality in schizophrenia patients and potentially other psychiatric disorders. In addition, the outcome of long-term SEP-363856 treatment on REM sleep remains to be investigated, as well as the effects on REM sleep recovery/rebound, and on REM sleep homeostasis in general.

Earlier work has shown that agonism at TAAR1 and 5-HT_1A_ receptors contributes to the mechanism of action of SEP-363856^2^. Consequently, the effects of SEP-363856 on REM sleep are in line with prior reports of REM suppression following treatment with selective TAAR1 agonists in mice, rats^8,13,14,16^ and nonhuman primates^15^. Although more robust effects on REM suppression were reported with partial (versus a full) TAAR1 agonist in rodents^13^^,16^, additional studies with full agonists are needed to better understand these discrepancies. TAAR1 partial agonists also promote wakefulness and reduced NREM sleep^8,13–16^. SEP-363856, which is a full agonist at TAAR1, has been shown to increase cumulative wake time in rats without altering cumulative NREM sleep^2^. In contrast, no changes in total sleep time, and an increase in NREM stages 2 and 3 were observed in healthy male subjects in the current study. Given that polyphasic sleep/wake cycles in rodents compared to the consolidated sleep periods in humans, differences in certain sleep EEG measures are to be expected across species. Interestingly, TAAR1 agonists reduced cataplexy in two mouse models of narcolepsy, a sleep disorder characterized by excessive sleepiness and REM sleep abnormalities^16^. Although these represent promising results, future clinical trials are required to determine the utility of TAAR1 agonists in the treatment of narcolepsy. In addition to TAAR1 agonism, activation of 5-HT_1A_ receptors likely also contributes to SEP-363856’s effects on sleep EEG. Several studies have reported REM suppression with 5-HT_1A_ agonists in healthy human subjects^18–22^. The 5-HT_1A_ receptor agonist eptapirone has been shown to increase wake time and sleep onset latency, with greater effects when administered in the morning compared to nighttime, while its REM suppression effects were only observed with nighttime administration^22^. In the current study, SEP-363856 was administered at nighttime which may have contributed to its REM-suppressing effect while minimizing the potential for 5-HT_1A_-mediated wake-promoting effects. In addition, Seifritz and colleagues^27^ observed enhanced EEG slow-wave sleep (defined as NREM stages 3/4) upon administration of low doses of the 5-HT_1A_ agonist ipsapirone in humans. However, opposite, or mixed results of 5-HT_1A_ agonist effects on NREM sleep are reported in other clinical and preclinical studies^19,22,28^. The increase in NREM sleep (stage 2 and 3) observed at 50 mg SEP-363856 could partially be modulated by 5-HT_1A_ receptors and potentially reflects a compensatory shift in sleep stages in response to the robust REM suppression. Slow-wave sleep is involved in the overnight consolidation of declarative memories and has been suggested to facilitate several cognitive processes^29–31^. Thus, treatments that enhance slow-wave sleep could have significant therapeutic implications.

Although REM suppression is a sensitive indicator of objective central effects of psychoactive drugs, it is not specific to a particular pharmacological mechanism^32–34^. Compounds acting on cholinergic, serotonergic, and/or noradrenergic transmission have all been shown to suppress REM sleep across several species. This includes most antidepressants (selective serotonin reupdate inhibitors (SSRIs), selective norepinephrine reupdate inhibitors (SNRIs), serotonin and norepinephrine reuptake inhibitors (SNRIs), tricyclic antidepressants and monoamine oxidase inhibitors), muscarinic antagonists, selective 5-HT_1A_ agonists, antihistaminic agents (e.g. promethazine) and TAAR1 agonists^8,15,22,33–37^. Although the associations with REM sleep reduction seem strongest for antidepressants, the value of this as an indicator of antidepressant efficacy is limited, especially for novel mechanisms. The sleep EEG profile of antidepressants, and particularly the effects on REM sleep are related to their ability to enhance noradrenergic and serotonergic transmission. Interestingly, SEP-363856 does not enhance monoamine release in mice^2^, further suggesting that its REM-suppressing effects occur through differential modulation of monoaminergic circuits, likely driven by activation of TAAR1 and/or 5-HT_1A_ receptors. Assessment of sleep microarchitecture, including changes in EEG spectral power, could aid in determining mechanism-specific distinctions of pharmacological effects on sleep. Although this study only assessed acute effects of SEP-363856 administration, the current results and its unique mechanism of action make it a potentially promising candidate for the treatment of sleep disorders characterized by abnormal REM sleep including REM sleep behavioral disorder (RBD), narcolepsy and obstructive sleep apnea (OSA). RBD is parasomnia characterized by repeated episodes of dream enactment behavior and REM sleep without atonia (RSWA) during PSG recordings. Both RSWA and RBD can also occur secondary to narcolepsy, obstructive sleep apnea, alpha-synucleopathies and in the setting of certain medications^38–43^. Thus, further investigation into the potential effects of SEP-363856 on RSWA is warranted.

Overall, the results of the current study indicate that a single dose of 50 mg of SEP-363856 potently suppressed REM sleep without altering total sleep time. The 10 mg dose of SEP-363856 also increased latency to REM sleep, but to a lesser extent. Dose-proportional increases in SEP-363856 AUC(0-8 h) correlated with changes in REM sleep. Both the 10 mg and 50 mg doses of SEP-363856 were generally safe and well tolerated. Consequently, REM sleep suppression associated with SEP-363856 represents a useful benchmark of dose, time, and concentration-dependent CNS activity.
